# Melatonin: Current Status and Future Perspectives in Plant Science

**DOI:** 10.3389/fpls.2015.01230

**Published:** 2016-01-11

**Authors:** Muhammad A. Nawaz, Yuan Huang, Zhilong Bie, Waqar Ahmed, Russel J. Reiter, Mengliang Niu, Saba Hameed

**Affiliations:** ^1^Key Laboratory of Horticultural Plant Biology, College of Horticulture and Forestry Sciences, Huazhong Agricultural University, Ministry of EducationWuhan, China; ^2^Department of Horticulture, University College of Agriculture, University of SargodhaSargodha, Pakistan; ^3^Sector Advisor-Horticulture, USAID-CNFALahore, Pakistan; ^4^Department of Cellular and Structural Biology, University of Texas Health Science Center at San AntonioSan Antonio, TX, USA

**Keywords:** melatonin, biosynthesis, physiological functions, antioxidants, root growth, stress tolerance

## Abstract

Melatonin (*N*-acetyl-5-methoxytryptamine) is a ubiquitous molecule with pleiotropic actions in different organisms. It performs many important functions in human, animals, and plants; these range from regulating circadian rhythms in animals to controlling senescence in plants. In this review, we summarize the available information regarding the presence of melatonin in different plant species, along with highlighting its biosynthesis and mechanisms of action. We also collected the available information on the effects of melatonin application on commercially important crops to improve their growth and development. Additionally, we have identified many new aspects where melatonin may have possible roles in plants, for example, its function in improving the storage life and quality of fruits and vegetables, its role in vascular reconnection during the grafting process and nutrient uptake from roots by modifying root architecture. Another potentially important aspect is the production of melatonin-rich food crops (cereals, fruits, and vegetables) through combination of conventional and modern breeding approaches, to increase plant resistance against biotic and abiotic stress, leading to improved crop yields, and the nutraceutical value of produce to solve food security issues.

## Introduction

Melatonin (*N-acetyl*-5-methoxytryptamine) is a widely studied bio-molecule and its function has been investigated in bacteria, mammals, birds, amphibians, reptiles, fish, and plants. It is a low molecular weight molecule, ubiquitously present with pleiotropic biological activities (Hardeland et al., 2011). Melatonin was discovered in bovine pineal gland in 1958 (Lerner et al., 1958). After its discovery, for the subsequent four decades it was considered exclusively as an animal hormone, especially a neurohormone (Reiter, 1991). In 1993, melatonin was possibly identified in the Japanese morning glory (*Pharbitis nil*) but these data were not reported until 1995 (Van-Tassel et al., 1995). Also in this year, the presence of melatonin in a number of edible plants was unequivocally demonstrated (Dubbels et al., 1995; Hattori et al., 1995). However, even prior to its identification in plants, melatonin had been shown to have effects on endosperm cells of the amaryllidacean *Scadoxus multiflorus* (syn. *Haemanthu skatherinae*) and on the epidermal cells of *Allium cepa* (Jackson, 1969; Banerjee and Margulis, 1973). Currently, research on plant melatonin is in an exponential growth phase and its functions in numerous plants have been uncovered; the number of publications related to plant melatonin has rapidly increased within the last decade (Arnao and Hernández-Ruiz, 2015; Reiter et al., 2015).

In animals, melatonin has many physiological roles including regulating circadian rhythms, mood, sleep, body temperature, bone metabolism, seasonal reproduction, locomotor activity, food intake, retina physiology, and immune system regulation (Maronde and Stehle, 2007; Pandi-Perumal et al., 2008; Reiter et al., 2009; Hardeland et al., 2012; Carrillo-Vico et al., 2013; García et al., 2014; Maria and Witt-Enderby, 2014; Manchester et al., 2015; Vriend and Reiter, 2015). Irrespective of its proven functions in animals, melatonin also has a wide range of functions in plants such as the promotion of seed germination and seedling growth and influencing plant senescence [death (Arnao and Hernandez-Ruiz, 2014)]. In this review, we summarize the currently-available information related to biosynthesis of melatonin, mechanisms of action and occurrence, role and functions in higher plants. We also speculate on new potential aspects where melatonin may have possible functions in plants.

## Biosynthesis

**Figure 1:**
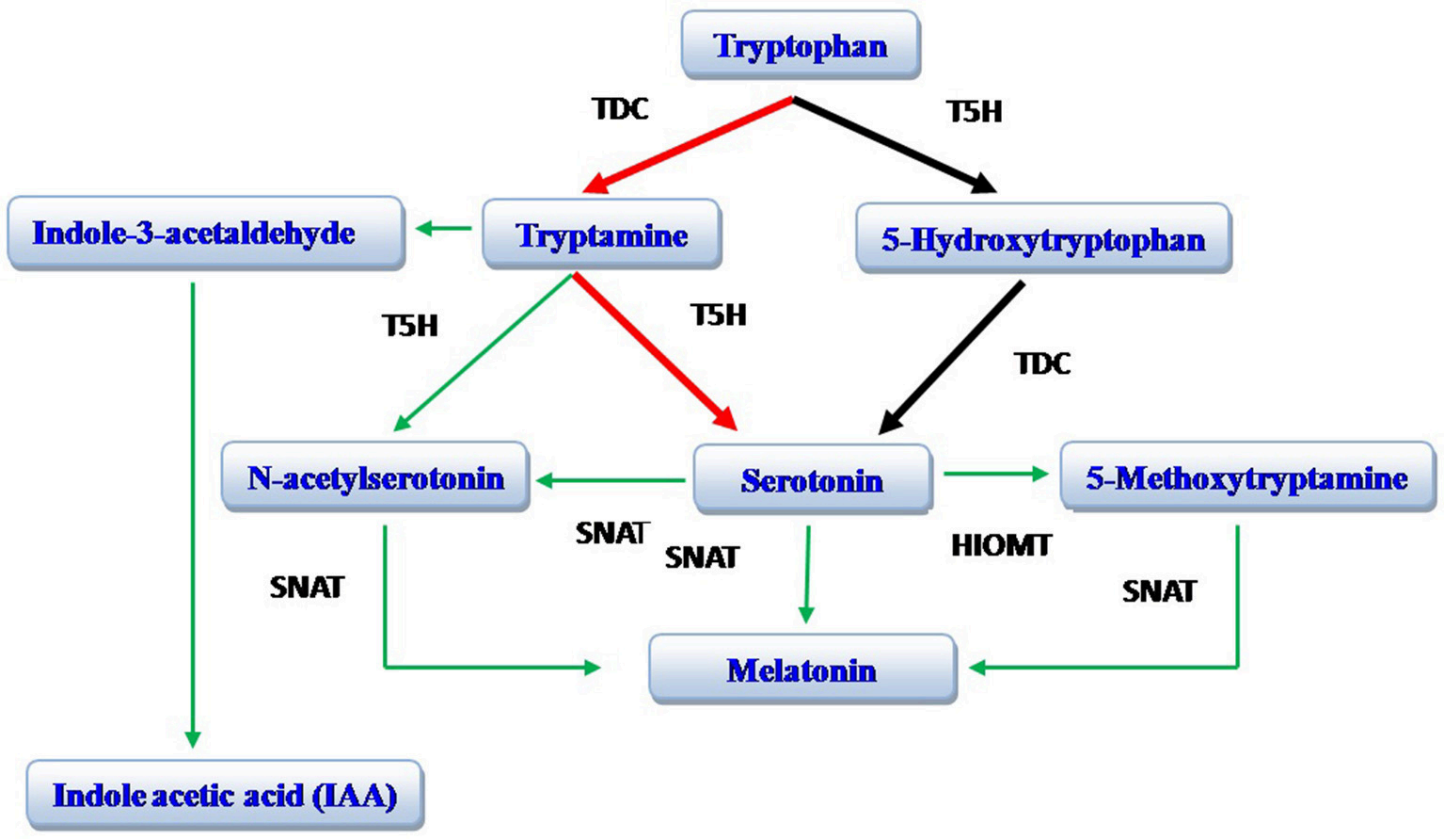
**Biosynthesis of melatonin**. The red arrows identify the preferred pathway in plants while the black arrows identify the major pathway in animals. TDC, tryptophan decarboxylase; T5H, tryptophan 5-hydroxylase; SNAT, serotonin N-acetyltransferase; HOMT, hydroxyindole-*O*-methyltransferase [also known as acetyl serotonin methyl transferase (ASMT)]. Modified from Arnao and Hernandez-Ruiz (2014).

The biosynthetic pathway of melatonin (*N*-acetyl-5-methoxytryptamine) is well known in vertebrates (Reiter, 1991; Falcón et al., 2009). In these species, tryptophan (an amino acid) is converted to 5-hydroxytryptophan; tryptophan 5-hydroxylase (T5H) is involved in this conversion. Thereafter, 5-hydroxytryptophan is converted to serotonin (Falcón et al., 2009). In animals, 5-hydroxytryptophan is the exclusive pathway for serotonin production in the pineal gland. In the plant St. John's Wort (*Hyericum perforatum* L.), 5-hydroxytryptophan is also involved in serotonin synthesis (Murch et al., 2000; Murch and Saxena, 2006). A recent study on rice, however, documents that the tryptamine pathway (tryptophan to tryptamine to serotonin) is more important in the production of serotonin (Park et al., 2012); this pathway has subsequently been found to be common to many plant species. Serotonin, in both plants and animals, is converted to N-acetyl serotonin catalyzed by serotonin N-acetyltransferase (SNAT), which is then methylated by hydroxyindole-*O*-methyltransferase (HIOMT; also known as acetyl serotonin methyl transferase, ASMT) resulting in the formation of melatonin. In plants (rice), N-acetyl serotonin is also directly produced from tryptamine and N-acetyltryptamine serves as an intermediate product; this pathway is catalyzed by SNAT and tryptophan 5-hydroxylase. Melatonin can be directly produced from serotonin with 5-methoxytryptamine serving as intermediate product in a process and catalyzed by HIOMT/ASMT and SNAT (Arnao and Hernandez-Ruiz, 2014; Byeon et al., 2014; Figure 1). Indole acetic acid (IAA) is also produced from tryptamine and indole-3-acetylaldehyde serves as an intermediate product (Krystyna et al., 2009; Arnao and Hernandez-Ruiz, 2014).

The concentration of melatonin in plants is much higher than levels in animals, and the biosynthesis of melatonin in plants also seems more complicated than in animals (Arnao and Hernandez-Ruiz, 2014). Due to more limited availability of information on the biosynthesis of melatonin and related products in plants, the definitive pathways await final definition (Byeon et al., 2015). Recently, transgenic rice has been shown to have genes for all the enzymes involved in the biosynthesis of melatonin (Byeon et al., 2014), and it is expected that soon the biosynthetic pathway will be described in detail. As far as the degradation of melatonin is concerned, N^1^-acetyl-N^2^-formyl-5-methoxykynuramine (AFMK) has been shown to be a secondary metabolite in vascular plants due to its enzymatic or non-enzymatic conversion from melatonin (Tan et al., 2007a). Recent studies on rice document that other metabolites of melatonin include 2-hydroxymelatonin (99%) and, to a much lesser extent, 4-hydroxymelatonin (0.05%) (Byeon et al., 2015).

## Melatonin: Presence in higher plants

Melatonin is found in a large number of plant species (Table 1). The roots, seeds, leaves, bulbs, and flowers were found to be rich sources of melatonin in most of the plant species examined. The roots of Huang-qin (*Scutellaria biacalensis*), which belongs to the family *Lamiaceae*, are an especially rich source of melatonin (7110 ng/g) (Reiter and Tan, 2002). Most of the plant species in which the presence of melatonin has been reported belong to the families *Rosaceae, Vitaceae, Poaceae, Apiaceae*, and *Brassicaceae*; however, the plants from some other families have also been shown to possess melatonin in large amounts. There is possibility that yet unstudied plant species may contain even higher concentrations of melatonin than have been reported. The concentration of melatonin in plants is affected by the genotype, environmental factors (photoperiod, temperature), stage of development (Zhao et al., 2012; Byeon and Back, 2014a; Table 1) and method of determination (Feng et al., 2014). This latter factor may be a major issue in the reports of melatonin in plants. The concentration of melatonin can vary among the different cultivars of a same species. Wang et al. (2009) quantified the level of melatonin in 58 and 25 different varieties of corn and rice, respectively, grown at same geographical location (Institute of Rice, Fujian Academy of Agricultural Sciences, Fuzhou, China), and observed huge variations in melatonin concentrations. The values of melatonin ranged from 11 to 2034 ng/g and 11 to 264 ng/g in corn and rice, respectively. These massive differences in concentrations suggest that the melatonin levels are determined, in large part, by the genotype of plants or the method used for melatonin quantitation.

**Table 1:** **Reported levels of melatonin in plants**.

**Common name**	**Scientific name**	**Family**	**Concentration (ng/g)**	**References**
**SEEDS**				
Milk thistle	*Silybum marianum*	Asteraceae	2	Reiter and Tan, 2002
Poppy	*Popaver somniferum*	Papaveraceae	6	Reiter and Tan, 2002
Anise	*Pimpinela anisum*	Apiaceae	7	Reiter and Tan, 2002
Coriander	*Coriandrum sativum*	Apiaceae	7	Reiter and Tan, 2002
Celery	*Apium graveolens*	Apiaceae	7	Reiter and Tan, 2002
Flax	*Linum usitatissimum*	Linaceae	12	Reiter and Tan, 2002
Green cardamom	*Elettaria cardamomum*	Zingiberaceae	15	Reiter and Tan, 2002
Alfalfa	*Medicago sativa*	Fabaceae	16	Reiter and Tan, 2002
Fennel	*Foeniculum vulgare*	Apiaceae (Umbelliferae)	28	Reiter and Tan, 2002
Sunflower	*Helianthus annuus*	Asteraceae	29	Reiter and Tan, 2002
Fenugreek	*Trigonella foenum-graecum*	Fabaceae	43	Reiter and Tan, 2002
Wolf berry	*Lycium barbarum*	Solanaceae	103	Reiter and Tan, 2002
Black mustard	*Brassica nigra*	Brassicaceae	129	Reiter and Tan, 2002
White mustard	*Brassica hirta*	Brassicaceae	189	Reiter and Tan, 2002
Barley	*Hordeum vulgare* L.	Poaceae	0.4, 0.87	Hattori et al., 1995; Badria, 2002
Rice (different varieties)	*Oryza sativa* japonica L.	Poaceae	1, 1.50, 11–234	Hattori et al., 1995; Badria, 2002; Wang et al., 2009
Oat	*Avena sativa* L.	Poaceae	2	Hattori et al., 1995
Corn (different varieties)	*Zea mays* L.	Poaceae	2, 1.88, 11–2034	Hattori et al., 1995; Badria, 2002; Wang et al., 2009
Tall fescue	*Festuca arundinacea*	Poaceae	5	Hattori et al., 1995
Huang-qin	*Scutellaria biacalensis*	Lamiaceae	7	Manchester et al., 2000
Almond	*Prunus amygdalus* Batsch	Rosaceae	39	Manchester et al., 2000
**GERMINATED SEEDS**				
Alfalfa	*Medicago sativa*	Fabaceae	0.133	Aguilera et al., 2015
Lentil	*Lens sculenta*	Fabaceae	0.217	Aguilera et al., 2015
Mung bean	*Vigna radiata*	Fabaceae	0.166	Aguilera et al., 2015
Onion	*Allium cepa*	Amaryllidaceae	0.302	Aguilera et al., 2015
Broccoli	*Brassica oleraceae*	Brassicaceae	0.439	Aguilera et al., 2015
Red cabbage	*Brassica oleraceae capitata rubra*	Brassicaceae	0.857	Aguilera et al., 2015
Radish	*Raphanu ssativus japonicum, rambo, sinicum rosae*	Brassicaceae	0.536	Aguilera et al., 2015
**LEAVES**				
St. John's wort	*Hypericum perforatum*	Hypericaceae	1750	Murch and Saxena, 2006
Fever few, gold	*Tanacetum parthenium*	Asteraceae	1920	Murch and Saxena, 2006
Fever few, green	*Tanacetum parthenium*	Asteraceae	2450	Murch and Saxena, 2006
Morning glory	*Pharbitis choisy*	Convolvulaceae	0.0005	Van-Tassel et al., 2001
Chine cabbage	*Brassica chinensis*	Brassicaceae	0.10	Hattori et al., 1995
Cabbage	*Brassica oleracea* L. var. *capitata*	Brassicaceae	0.10, 0.30	Hattori et al., 1995; Badria, 2002
Tomato (Transgenic)	*Solanum lycopersicum* L.	Solanaceae	45	Wang et al., 2014
Lupin	*Lupinus albus* L.	Fabaceae	75.6	Arnao and Hernández-Ruiz, 2013
**SHOOTS**				
Morning glory	*Pharbitis choisy*	Convolvulaceae	0.004	Van-Tassel et al., 2001
Asparagus	*Asparagus officinalis* L.	Asparagaceae	0.01	Hattori et al., 1995
Red pigweed	*Chenopodium rubrum* L.	Chenopodiaceae	0.20	Kolar et al., 1997
**FLOWERS**				
St. John's wort	*Hypericum perforatum*	Hypericaceae	4390	Murch and Saxena, 2006
**FRUITS**				
Banana	*Musa paradisiaca* L.	Musaceae	0.47	Dubbels et al., 1995
Banana	*Musa ensete*	Musaceae	0.66	Badria, 2002
Banana	*Musa sapientum* L.	Musaceae	0.01	Arnao and Hernández-Ruiz, 2013
Cucumber	*Cucumis sativus* L.	Cucurbitaceae	0.03, 0.59	Hattori et al., 1995; Badria, 2002
Pineapple	*Ananas comosus* (L.) Meri.	Bromeliaceae	0.04, 0.28, 0.30	Hattori et al., 1995; Badria, 2002; Arnao and Hernández-Ruiz, 2013
Apple	*Malus domestica* (Borkh)	Rosaceae	0.05, 0.16	Hattori et al., 1995; Badria, 2002
Tomato	*Solanum lycopersicum* L.	Solanaceae	0.5, 0.30	Dubbels et al., 1995; Badria, 2002
Tomato (dry weight basis)	*Solanum lycopersicum* L.	Solanaceae	7.5–250	Riga et al., 2014
Chilies (dry weight basis)	*Capsicum annum* L.	Solanaceae	31–93	Riga et al., 2014
Cherry	*Prunus cerasus* L.	Rosaceae	18.0	Burkhardt et al., 2001
Kiwifruit	*Actinidia chinensis*	Actinidiaceae	0.02	Hattori et al., 1995
Pomegranate	*Punica granatum* L.	Lythraceae	0.17	Badria, 2002
Barbera grape (skin)	*Vitis vinifera* L.	Vitaceae	0.63	Iriti et al., 2006
Croatina grape (skin)	*Vitis vinifera* L.	Vitaceae	0.87	Iriti et al., 2006
Cabernet franc grape (Skin)	*Vitis vinifera* L.	Vitaceae	0.01	Iriti et al., 2006
Cabernet sauvignon grape (Skin)	*Vitis vinifera* L.	Vitaceae	0.42	Iriti et al., 2006
Marzemino grape (skin)	*Vitis vinifera* L.	Vitaceae	0.03	Iriti et al., 2006
Nebbiolo grape (skin)	*Vitis vinifera* L.	Vitaceae	0.97	Iriti et al., 2006
Sangiovese grape (skin)	*Vitis vinifera* L.	Vitaceae	0.33	Iriti et al., 2006
Merlot grape (skin)	*Vitis vinifera* L.	Vitaceae	0.26	Iriti et al., 2006
Sangiovese grape	*Vitis vinifera* L.	Vitaceae	1.50	Mercolini et al., 2012
Albana grape	*Vitis vinifera* L.	Vitaceae	1.20	Mercolini et al., 2012
Burlat cherry	*Prunus avium* L.	Rosaceae	0.22	Gonzalez-Gomez et al., 2009
Sweetheart cherry	*Prunus avium* L.	Rosaceae	0.06	Gonzalez-Gomez et al., 2009
Pico Negro cherry	*Prunus avium* L.	Rosaceae	0.12	Gonzalez-Gomez et al., 2009
Navalinda cherry	*Prunus avium* L.	Rosaceae	0.03	Gonzalez-Gomez et al., 2009
Van cherry	*Prunus avium* L.	Rosaceae	0.01	Gonzalez-Gomez et al., 2009
Pico Colorado cherry	*Prunus avium* L.	Rosaceae	0.05	Gonzalez-Gomez et al., 2009
Hongdeng cherry	*Prunus avium* L.	Rosaceae	35.6	Badria, 2002
Rainier cherry	*Prunus avium* L.	Rosaceae	124.7	Badria, 2002
Tart cherry (Balaton)	*Prunus cerasus*	Rosaceae	22.90	Burkhardt et al., 2001; Kirakosyan et al., 2009
Tart cherry (Montmorency)	*Prunus cerasus*	Rosaceae	15, 12.30	Burkhardt et al., 2001; Kirakosyan et al., 2009
Wild strawberry	*Fragaria ananassa* Duch.	Rosaceae	0.01	Hattori et al., 1995
Camarosa strawberry	*Fragaria ananassa* Duch.	Rosaceae	5.58	Sturtz et al., 2011
Candonga strawberry	*Fragaria ananassa* Duch.	Rosaceae	5.50	Sturtz et al., 2011
Festival strawberry	*Fragaria ananassa* Duch.	Rosaceae	11.26	Sturtz et al., 2011
Primoris strawberry	*Fragaria ananassa* Duch.	Rosaceae	8.50	Sturtz et al., 2011
Orange	*Citrus sinensis* Osbeck.	Rutaceae	0.15	Johns et al., 2013
Mango	*Mangifera indica* L.	Anacardiaceae	0.70	Johns et al., 2013
Papaya	*Carica papaya* L.	Caricaceae	0.24	Johns et al., 2013
Walnut	*Juglans regia* L.	Juglandaceae	3.5	Reiter et al., 2005
**COLEOPTILES**				
Canary grass	*Phalaris canariensis* L.	Poaceae	26.7	Hernández-Ruiz et al., 2005
Wheat	*Triticum aestivum* L.	Poaceae	124.7	Hernández-Ruiz et al., 2005
Barley	*Hordeum vulgare* L.	Poaceae	82.3	Hernández-Ruiz et al., 2005
Oat	*Avena sativa* L.	Poaceae	90.6	Hernández-Ruiz et al., 2005
**ROOTS**				
Beet	*Beta vulgaris* L.	Amaranthaceae	0.01	Dubbels et al., 1995
Carrot	*Daucus carota*	Apiaceae	0.06, 0.49	Hattori et al., 1995; Badria, 2002
Ginger	*Zingiber officinale* (Roscoe)	Zingiberaceae	0.6, 1.42	Hattori et al., 1995; Badria, 2002
Red radish	*Raphanus sativus* L.	Brassicaceae	0.6	Hattori et al., 1995
Radish	*Raphanus sativus* L.	Brassicaceae	0.76	Badria, 2002
Turnip	*Brassica compestris* L.	Brassicaceae	0.7, 0.50	Hattori et al., 1995; Badria, 2002
Lupin	*Lupinus albus*L.	Fabaceae	55.6	Arnao and Hernández-Ruiz, 2013
Huang-qin	*Scutellaria biacalensis*	Lamiaceae	7110	Reiter and Tan, 2002
**BULBS**				
Onion	*Allium cepa* L.	Amaryllidaceae	0.03, 0.29	Hattori et al., 1995; Badria, 2002
Garlic	*Allium sativum* L.	Amaryllidaceae	0.58	Badria, 2002

*The plants having more than one value for melatonin concentration are reported from different sources, and references are given in sequence*.

Melatonin biosynthesis in rice seedlings is enhanced when they are exposed to high temperatures or are maintained under dark conditions. Melatonin concentration was increased from 2.95 to 4.9 ng/g when rice seedlings were exposed to darkness at 55°C. The increase in melatonin level was associated with the enhanced activity of both SNAT and HIOMT/ASMT, both of which are involved in the biosynthetic pathway of melatonin (Byeon and Back, 2014a). Similarly, tomato plants exposed to shade exhibited an increase in the levels of melatonin (135%) (Riga et al., 2014). In sweet cherry, the endogenous levels of melatonin exhibited two obvious peaks, one at 05:00 h and a second at 14:00 h (Zhao et al., 2012); the first peak was attributed to darkness while the second peak may have been related to high temperature or high light intensity (stressful conditions). The enzyme kinetic analyses show that SNAT and HIOMT/ASMT enzymes exhibited thermophilic features (Byeon et al., 2014); purified recombinant SNAT and HIOMT/ASMT have shown optimal activities at 55 and 45°C, respectively (Byeon et al., 2013). Such high temperatures (45–55°C), however, are not typically experienced under natural environmental conditions.

In many edible fruits the presence of melatonin has also been reported (Table 1). In these species, endogenous levels of melatonin also vary according to cultivars studied, and with the stage of fruit development (Zhao et al., 2012; Feng et al., 2014). In sweet cherries, the first stage of fruit development is associated with low melatonin levels in both cultivars (Hongdeng and Rainier) (15 ng/g): during the second stage, these values exhibit large increases (36.6 and 124.7 ng/g, respectively) and in the third stage, the values drop to 10–20 ng/g. In most fruits, during the second stage of fruit development, cell elongation and cell expansion, and embryo and seed development occurs. So a possible role of melatonin in fruit development cannot be precluded; however, this still needs to be verified.

In a recent study it was reported that seed germination is associated with marked increases in the concentration of melatonin (2–3 fold); this suggested to the authors that the germinated seeds of the edible species may have utility as a food to raise the melatonin levels in plasma (Aguilera et al., 2015). Plants have also been genetically engineered to produce increased levels of melatonin compared to their wild types. Thus, Kang et al. (2010) observed that rice transgenic lines overexpressing human SNAT increased the biosynthesis of melatonin when compared with wild type rice plants. Also, in a recent study *oAANAT* and *oHIOMT* genes, encoding key enzymes catalyzing the last two steps in melatonin biosynthesis were introduced into the Micro-Tom tomato from the pineal gland of *Ovis aries* (sheep). The melatonin contents of the Micro-Tom tomato transgenic lines were higher compared to their wild type, indicating the transferred animal genes were functional in the biosynthesis of melatonin in plants (Wang et al., 2014). Similarly, melatonin-rich transgenic rice plants overexpressing sheep SNAT have significantly higher levels of melatonin than in wild type rice (Park and Back, 2012). Clearly, the biosynthesis of melatonin in plant species can be altered by the introduction of genes from vertebrates; such genetically-altered plants may have utility as food because of their induced resistance against diseases, and to increase the yield, quality and nutritional value of crops.

In addition to plants listed in Table 1, Chen et al. (2003) measured melatonin levels in 64 commonly used medicinal herbs; they found concentrations of melatonin ranging from 12 to 3771 ng/g. Also, exceptionally high melatonin concentrations (227–233 μg/g) have been found in four different varieties of Pistachio (*Pistacia vera* L.) (Oladi et al., 2014); they are the highest values reported for any plant organ to date.

Melatonin in plants has been detected by several methods including radioimmunoassay (RIA), enzyme-linked immunosorbent assay (ELISA), gas chromatography-mass spectrometry (GS-MS), and high-performance liquid chromatography (HPLC) with electrochemical detection (HPLC-ECD), fluorescence detection (HPLC-FD), or HPLC-MS. These methods differ in their sensitivity and specificity (Feng et al., 2014). Kolár and Machácková (2005) suggested that RIA is not a reliable method for melatonin detection in plant samples, since the measurements have not been validated by other methods. For each method, different extraction solvents were employed. Oladi et al. (2014) measured melatonin using GC-MS, while for melatonin extraction an ultrasound-assisted solid-liquid extraction method was used. They found that the type of solvent, volume of solvent, temperature, sonication time and pH influenced extraction efficacy. Under optimized conditions they found highest ever reported melatonin levels from Pistachio (*Pistacia vera* L.) compared to any other plant in which melatonin was estimated. So the detection method might be the source of variation in melatonin concentrations among plant species. This issue needs to be addressed.

## Role in plants

Melatonin has proven to be ubiquitously synthesized in plant organs (Park et al., 2012; Byeon et al., 2013; Byeon and Back, 2014a,b; Byeon et al., 2014; Wang et al., 2014). Pleiotropic roles ranging from enhancing germination to delaying senescence of plants have been reported (Kolár and Machácková, 2005; Arnao and Hernández-Ruiz, 2006; Krystyna et al., 2009; Tan et al., 2012; Chan and Shi, 2015; Wei et al., 2015). While melatonin's role have highlighted the modulation of circadian rhythms in mammals (Bonnati-Carrion et al., 2014; Hardeland, 2015; Vriend and Reiter, 2015), this function has not been thoroughly examined in plants (Kolar et al., 1997). Thus, this subject is not discussed below; rather the data summarized below primarily consider the functions of melatonin in enhancing growth and preserving the integrity of plants under stressful conditions.

### Propagation

*In vitro* germplasm storage via cryopreservation is an effective tool to ensure conservation of tree species, but plant cells and tissues are exposed to multiple stresses including osmotic injury, desiccation and low temperature injury during the cryopreservation process; this contributes to problems during the regrowth of cryopreserved materials (Uchendu et al., 2013). Supplementing both preculture and regrowth media with melatonin (0.1–0.5 μM melatonin for 24 h) significantly enhanced regrowth of frozen shoots compared with the untreated shoots (Uchendu et al., 2013). Similarly, 0.1 μM melatonin as pre-cryopreservation treatment to callus of *Rhodiola crenulata* (endangered plant species) also improved their recovery (Zhao et al., 2011).

Seed treatment with 100 μM melatonin for 12 h significantly improved the percentage germination of cucumber seeds (Zhang et al., 2013). Low concentrations of melatonin (1 μM) enhanced the germination rate of cucumber under salinity stress by regulating the biosynthesis and catabolism of abscisic acid (ABA) and gibberellic acid (GA_4_) (Zhang et al., 2014). Cuttings are also used as a means of propagation for many commercially important horticultural crops. The exogenous application of melatonin to roots of grape cuttings improved their growth by enhancing water stress tolerance. It increased the activity of antioxidant enzymes and the activities of non-enzymatic antioxidants; melatonin treatment also kept the internal lamellar system of chloroplasts well preserved and reduced ultrastructural destruction caused by drought stress (Jiang et al., 2014). A 2–3 fold rise in seed germination rate is common when they are treated with melatonin (Aguilera et al., 2015). While melatonin has been shown consistently to elevate the germination rate of seeds, the mechanisms of this stimulatory action remain to be identified.

### Growth and development

Several studies have noted that melatonin regulates these physiological functions of plants; melatonin generally improves the growth of roots, shoots and explants (Murch et al., 2001; Hernández-Ruiz et al., 2005). The initial report of the direct involvement of melatonin in stimulating plant growth was reported in 2005 by Hernández-Ruiz et al. (2005); they observed that melatonin extended the coleoptiles (10–55%) of canary grass, wheat, barley and oat (monocots). Later, it was found that 0.5–1 μM application of melatonin enhanced the initial seminal root length, growth and root biomass of transgenic rice plants (Park and Back, 2012). Melatonin is now known to alter many plant characteristics including germination (Zhang et al., 2014), seedling growth, alteration of flowering time, grain yields, and senescence (Wang et al., 2013a,b; Byeon and Back, 2014b). Somewhat unexpectedly, Byeon and Back (2014b) found that melatonin increased early seedling growth, but delayed flowering and reduced grain yield in transgenic rice over expressing sheep SNAT.

In animals, melatonin has also been reported to have anti-aging actions by delaying senescence (Acuna-Castroviejo et al., 2011; Rosales-Corral et al., 2012; Hardeland, 2013; Reiter et al., 2014). Long term soil application of 100 μM melatonin also altered the metabolic status and delayed protein degradation in the apple plant (*Malus hupehensis* Rehd.), increased the chlorophyll content, the photosynthetic rates, and photosynthetic end products (sucrose, sorbitol, and starch) compared to control plants (Wang et al., 2013b). These changes were all associated with better protein preservation capacity. Consistent with this, Wang et al. (2013a), reported that long term application of 100 μM melatonin to “Hanfu” apple (*Malus domestica* Borkh.) delayed drought-induced leaf senescence by reducing oxidative stress and suppressing the up-regulation of *senescence-associated gene 12 (SAG-12)* and *pheophorbide a oxygenase (PAO)*. Exogenous melatonin application also delayed natural leaf senescence in *Arabidopsis* (Shi et al., 2015a). Post-harvest losses of fruits and vegetables are very high (20–40%); according to estimates of the Food and Agricultural Organization (FAO), 32% of the all food produced in 2009 was wasted or lost (Lipinski et al., 2013). Considering the delay of aging and senescence due to pre and post-application of melatonin, it is possible that melatonin may play an important role in extending the shelf life of fruits and vegetables, and prove helpful in “on tree storage” (fruit crops), both of which may decrease post-harvest losses of fresh horticultural commodities.

Zhao et al. (2012) observed that in sweet cherry the concentrations of melatonin are comparatively lower in the first and third stages of fruit development while being much higher during the second stage of fruit development. During the second stage, cell elongation, cell expansion and embryo and seed development occurs, so the possible involvement of melatonin in these processes is clearly likely; however, additional investigations are required to precisely define the mechanisms by which melatonin influences development of fruits and vegetable crops. A recent study provides direct evidence that seed coating with melatonin significantly increased the leaf area, plant height, pods per plant, seeds per plant, and fatty acid contents of soybean plants (Wei et al., 2015). This study suggests new avenues to enhance crop yields. Seeds coated with melatonin could be potentially used for a large number of commercially important agronomic and horticultural crops. This has the potential to revolutionize the seed industry.

### Stress tolerance

#### Salinity

Salinity is a major environmental factor that limits crop growth and productivity; it leads to huge economic losses worldwide (Allakhverdiev et al., 2000). Salinity not only induces water deficit caused by osmotic stress, it also disturbs key biochemical process (photosynthesis, protein synthesis, energy, and lipid metabolism) in plant cells (Allakhverdiev et al., 2000; Li et al., 2012). Plants use various strategies to cope with these stressors; these involve the exclusion of selective ions, ion compartmentalization, synthesis of compatible solutes, alterations in the photosynthetic pathway, changes in membrane structure, induction of antioxidant enzymes, stimulation of phytohormones and regulation of gene expression (Parida and Das, 2005). Recently, Arnao and Hernandez-Ruiz (2014) reviewed the auxin-independent effects of melatonin as a plant growth regulator in various plant species. Exogenous application of melatonin (0.1 μM) significantly alleviated the growth inhibition caused by elevated salinity; this enabled the plants to maintain their photosynthetic capacity. The application of melatonin also decreased the oxidative damage caused by ROS by directly scavenging H_2_O_2_ and enhancing the activities of antioxidant enzymes including ascorbate peroxidase, catalase, and peroxidase (Li et al., 2012). Salinity exerts its negative impact irrespective of growth stage of the plants, and its effects range from seed germination to plant senescence, and occur throughout the life cycle. Seed germination and plant growth is severely affected by saline stress (Ungar, 1996; Parida and Das, 2005; Li et al., 2012). In every case melatonin proved its importance by ameliorating the effects caused by salt stress and improved germination and plant growth. In cucumber (*Cucumis sativus* L.), pre-sowing seed treatment with melatonin (1 μM) enhanced the rate of germination and subsequent growth under 150 mM NaCl stress; this increase was accompanied by approximately a 5-fold elevation in antioxidant enzyme activities (superoxide dismutase, catalase, peroxidase; Zhang et al., 2014). Melatonin has also been found to be involved in the biosynthesis and catabolism of gibberellic (GA) and abscisic acids (ABA), respectively; it was shown to up-regulate ABA catabolism genes and down-regulate ABA biosynthesis genes resulting to a rapid reduction in ABA. At the same time, it positively up-regulated GA biosynthesis genes during the early stage of germination, which leads to better germination and better plant growth during the initial stages (Zhang et al., 2014). Melatonin application enhanced tolerance to salt and drought stress in soybean, and up-regulated the expression of genes that were inhibited by salt stress (Wei et al., 2015).

A recent study conducted using bermudagrass revealed that exogenous melatonin application conferred abiotic stress tolerance and it was observed that 3933 transcripts (2631 were up-regulated and 1572 were down-regulated) were differentially expressed compared to non-treated plants (Shi et al., 2015b). The genes involved in nitrogen metabolism, major carbohydrate metabolism, tricarboxylic acid (TCA)/org transformation, transport, hormone metabolism, metal handling, redox, and secondary metabolism were over expressed, clearly showing the involvement of melatonin in influencing metabolic activity.

#### Cold

Low temperature stress leads to significant damage to agricultural crops; low temperature alters plant physiology, biochemistry and molecular biology (Bajwa et al., 2014). Many scientists are working on the development of cold tolerant commercially-important crop cultivars. Recently, melatonin was shown to significantly alleviate cold stress in a number of plants. Melatonin treated (10–30 μM) *Arabidopsis thaliana* plants produced higher fresh weight, root length and plant height compared to untreated plants (Bajwa et al., 2014). Like other plants, low temperature damages wheat plants by reducing leaf area, leaf water content, photosynthetic pigment content, and the accumulation of ROS caused lipid peroxidation of membranes. The application of melatonin (1 mM for 12 h) to wheat seedlings increased the activity of the antioxidant enzymes, superoxide dismutase, guaiacol peroxidase, ascorbate peroxidase, and glutathione reductase leading to improved plant growth by reducing oxidative damage (Turk et al., 2014). More recently, it has been found that the exogenous application of melatonin increased salt, drought and cold resistance in bermudagrass (*Cynodon dactylon* L. Pers.). In this study, melatonin activated not only several antioxidants but also induced higher concentration of 54 secondary metabolites including amino acids, organic acids, sugars, and sugar alcohols (Shi et al., 2015b).

#### Heat stress

Extremes temperatures affect membrane fluidity and enzyme activities (Zhang et al., 2015) leading to alterations in growth and development patterns and yield losses. In plants under stressful conditions, the genes responsible for melatonin biosynthesis are typically activated resulting in higher levels of melatonin. As an example, under high temperature conditions the level of melatonin is increased in rice (Byeon and Back, 2014a) suggesting a role of melatonin in defense against heat stress. In green micro-algae *Ulva* sp. rise in temperature increases melatonin levels, confirming its ability to improve heat tolerance (Tal et al., 2011). Application of melatonin has the potential to reverse the inhibitory effect of light and high temperature on photosensitive and thermosensitive *Phacelia tanacetifolia* Benth seeds (Tiryaki and Keles, 2012). Melatonin application increased germination percentage of heat stressed *Arabidopsis thaliana* seeds up to 60% compared to control; this effect was likely due to powerful antioxidant activity of melatonin (Hernández et al., 2015). Similarly in another recent study, Shi et al. (2015b) reported that application of melatonin activated stress responsive genes in Bermuda grass. *C-REPEATBINDING FACTORS/DEHYDRATION-responsive ELEMENT-BINDING PROTEIN* (*CBF/DREB*) genes and target genes, heat shock transcription factors (TFs), zinc finger TFs, *WRKY, MYB, bHLH* genes, and hormone-related genes exhibited a 16-fold over expression compared to levels in control plants.

#### Drought, ultraviolet radiations, heavy metals, and chemicals stress

Melatonin has also proven its protective role against drought, ultraviolet radiation, heavy metals and chemicals stress. Transgenic Micro-Tom tomato plants overexpressing the homologous ovine *AANAT* and *HIOMT* genes exhibited loss of apical dominance and enhanced drought tolerance (Wang et al., 2014). Plant species sensitive to ozone damage have lower levels of melatonin compared to ozone resistant species (Dubbels et al., 1995). Similarly, Alpine and Mediterranean plant species growing in high UV-exposed natural habitats have higher levels of melatonin compared to their counterparts growing under low UV exposure areas (Simopoulos et al., 2005). Afreen et al. (2006) observed higher concentrations of melatonin in roots of *Glycyrrhiza uralensis* when exposed to UV-B radiation; they proposed elevated melatonin levels were protective against plants the augmented oxidative damage. Subsequently, Zhang et al. (2012) confirmed the protective role of melatonin against UV-B. When exposed to UV-B radiation, DNA damage was reduced in transgenic *Nicotiana sylvestris* plants expressing melatonin synthesis genes. Melatonin is also useful to save plants from heavy metals stress, as presowing seed treatment of red cabbage seed (*Brassica oleracea* rubrum) eliminated the toxic effects of copper ions (0.5 and 1 mM) during germination and early seedling growth (Posmyk et al., 2008). Similarly, Arnao and Hernández-Ruiz (2009) observed that application of zinc sulfate (1 mM) increased the concentration of melatonin up to 6-fold in barley (*Hordeum vulgare* L.) roots, suggesting the protective role of melatonin against chemical and other abiotic stressors. Melatonin has also be reported to provide protection against butafenacil (a singlet oxygen-generating herbicide), in the study in question, melatonin-rich transgenic rice plants exhibited lower levels of malondialdehyde and hydrogen peroxide. These plants also exhibited elevated superoxide dismutase and catalase activities compared to control plants (Park et al., 2013).

#### Disease resistance/control

Plant diseases cause major production and economic losses in agriculture worldwide, and both the public and private sectors are working to control the plant diseases through various strategies ranging from forecast and diagnosis of diseases to the production of disease resistant cultivars. In addition to many other positive functions in plants, exogenous application of melatonin (0.05–0.5 mM) improved resistance against one of the most severe diseases, *Marssonina* apple blotch (fungal diseases caused by *Diplocarpon Mali*); this involved modulating the activities of antioxidant enzymes and plant defense related enzymes (Yin et al., 2013). Also, Ishihara et al. (2008) observed that the activation of tryptophan pathway leads to establishment of effective physical defenses by enriching serotonin in rice leaves; serotonin suppresses the growth of fungal hyphae in leaf tissues. Although the authors did not mention the possible involvement of melatonin, this possibility cannot be ignored since serotonin is the precursor of melatonin in the biosynthetic pathway (Reiter, 1991; Falcón et al., 2009; Park et al., 2012; Arnao and Hernandez-Ruiz, 2014). In a recent study, the application of 10 μM melatonin on to *Arabidopsis* induced pathogenesis-related genes which further supports the idea that melatonin may be a defense signaling molecule in plants against pathogens (Lee et al., 2014). After compiling a recent review, the authors (Vielma et al., 2014) concluded that melatonin is an important therapeutic alternative to fight against bacterial, viral and parasitic infections in vertebrates (human, mammals, equine) as has been observed in plants. Certainly, the possibility that melatonin may help in controlling plant diseases (fungal, bacterial, viral, viroides) should not be overlooked and requires further investigation. Similarly, the potential role of melatonin in defense against insect attacks should be considered, as it has been reported that dopamine (a catecholamine) functions as a antiherbivore defense in temperate green alga *Ulvari aobscura* (Van Alstyne et al., 2006). Other secondary metabolites have also been isolated from plants which serve as juvenile hormone antagonists against insects and can be used to kill the insects at the larval stage (Lee et al., 2015). Thus, melatonin (an indoleamine) might have a role is defense against insect attack, and could prove to be a potential means to control or reduce insect feeding on commercial crops, as insects cause huge losses (billions of dollars) and substantially reduce crops yields (Boyer et al., 2012).

#### Phytoremediation

The water hyacinth grown under bright sunlight (10,000–15,000 μW/cm^2^) produces extremely high concentrations of melatonin and N^1^-acetyl-N^2^-formyl-5-methoxykynuramine (AMFK) as compared to plants grown in artificial light (400–450 μW/cm^2^) (Tan et al., 2007a). On the basis of these findings and others, the authors proposed that the presence of high concentrations of these molecules save these pollutant-resistant plants from the harsh environmental contaminants. They suggested that plants containing up-regulated melatonin and AMFK levels could be used for soil phytoremediation (Tan et al., 2007a). They further supported this when they found that soil application (5 μM) of melatonin improved the copper tolerance of pea (*Pisum sativum* L.) plants (Tan et al., 2007b). Thus, melatonin was found to be effective in preventing the death of pea plants grown in soil contaminated with copper. Moreover, melatonin itself is environmentally friendly. Other studies suggest the use of biotechnology and genetic engineering techniques to increase the phytoremediative potential of already existing plants used for this purpose (Dietz and Schnoor, 2001; Cherian and Oliveira, 2005; Lal and Srivastava, 2010; Behera, 2014). Thus, an integrated approach toward phytoremediation may lead to the desired results.

## Mechanisms of action

The mechanisms of action of melatonin is not clearly understood in plants; however, it modifies plant growth and development by acting as an antioxidant, membrane stabilizer, and by up and down regulating gene expression. Some of melatonin actions in plants may be receptor-mediated while others are receptor-independent. Recently, Arnao and Hernandez-Ruiz (2014) suggested that melatonin performs some of its functions in plants by actions similar to those of indole-3-acetic acid (IAA). However, in *Gonyaulax polyedra* (dinoflagellate) the first observable reaction to melatonin is a 90-fold increase in bioluminescence coupled with the release of H^+^ into the cytoplasm (Hardeland, 1993). In plants, this aspect is a missing link in understanding the biological functions of melatonin and it requires the attention of plant scientists.

## Future perspectives

Some successful examples of transgenic plants having introduced genes from vertebrates have shown substantially elevated concentrations of melatonin. In the future, additional transgenic crops will likely be produced, and the altered biosynthesis of melatonin in these plants may be used as a tool to induce resistance against biotic and abiotic stresses leading to increased crop yields. Also, considering the importance and therapeutic value of melatonin for humans, the pharmaceutical industry should capitalize on the potential human benefits of related pharmaceutical preparations for humans, animals and plants. Presumably, in the future we will see fruits and vegetables with higher levels of melatonin produced by the combination of conventional and modern breeding approaches. Few reports are available related to the possible role of melatonin in helping to control diseases and insects; this area of research should be aggressively explored with a definition of the specific defense mechanisms. This information could lead to the use of melatonin on a commercial scale. Melatonin enhances the phytoremediative capacity of plants, but further studies are required. Whether melatonin improves the phytoremediative capacity of the hyperaccumulator plant species would be important to document, and if so mechanism involved would require definition.

It has been widely reported that application of melatonin promotes root growth but the roles of melatonin in nutrient uptake still needs to be investigated; to date no reports have been published clarifying the interactions between melatonin and nutrient uptake and transport. Similarly, very limited information is available on the response of foliar applications of melatonin to its absorption and modification of plant growth and development.

Although melatonin is ubiquitously distributed in plants, it is not known whether all plant organs synthesize this indoleamine. Its mechanism of transport throughout the plant also must be explored.

As many scientists have observed the auxin-like activity of melatonin along with its pleiotropic functions (in animals and plants). Further detailed investigations on the possible role of melatonin in *in vitro* plant propagation, propagation through cutting, grafting and vascular reunion, flower development, enhancing male to female ratio in vegetables (cucurbits), improvement of fruit setting, fruit development, parthenocarpy, fruit drop (a major issue in commercially important fruit crops like citrus, mango, guava, etc.), role in breaking seed and tuber dormancy, fruit quality (size, color, nutraceutical value), seed development, fruit ripening and senescence (to improve post-harvest life/shelf life of fruits, vegetables, and cut flowers) needs clarification. Root treatment of melatonin may help to improve the success ratio and initial root development and growth of crops which require nursery transplanting (rice, tomato, chilies, cabbage, cauliflower, eggplant etc.). The role of melatonin in grafted plants also should be investigated; do different scion stock combinations affect the concentration of melatonin in roots and shoots alternatively?

**Figure 2:**
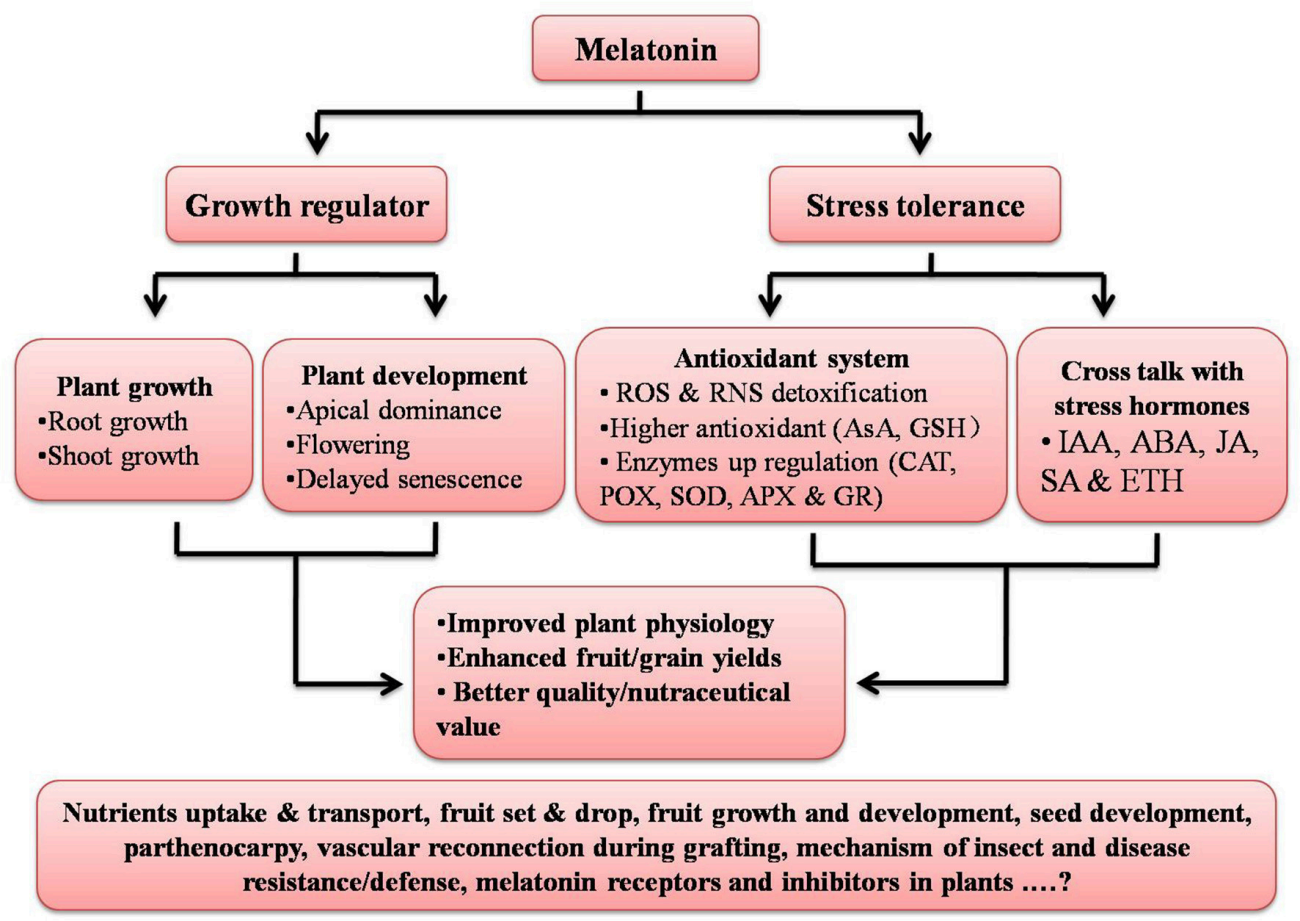
**Summary of functions of melatonin in plants**. Aspects given at base of figure still need to be explored. ROS, Reactive oxygen species; RNS, Reactive nitrogen species; AsA, Ascorbic acid; GSH, Glutathione; CAT, Catalase; POX, Peroxidase; SOD, Superoxide dismutase; APX, Ascorbate peroxidase; GR, Glutathione reductase; IAA, Indole-3-acetic acid; ABA, Abscisic acid; JA, Jasmonic acid; SA, Salicylic acid; and ETH, Ethylene.

Recently, scientists have documented an active role of auxin in grafting and vascular connection establishment (Melnyk et al., 2015); since melatonin has been reported to act like an auxin, its involvement with auxin in terms of the vascular reconnection should to be examined. While the physiological and biochemical roles of melatonin in plants are in part clarified, there is not a single study related to melatonin specific synthesis or action inhibitors or the presence or absence of melatonin receptors in plants; this is an area worthy of investigation (Figure 2). Keeping in mind the physiological, biochemical, and genetic and epigenetic actions of melatonin in multiple organisms, it seems melatonin may prove to be an important molecule to influence especially field crops, and may prove helpful in increasing crops yields and the nutraceutical value helping to address the food security issues around the world.

## Author contributions

MAN, YH, WA, MN, and SH wrote the manuscript, ZB and RR revised, and finally approved the manuscript for publication.

### Conflict of interest statement

The authors declare that the research was conducted in the absence of any commercial or financial relationships that could be construed as a potential conflict of interest. The reviewer Yong-Zhong Liu declares that, despite being affiliated with the same institute as the authors Muhammad A. Nawaz, Yuan Huang, Zhilong Bie, Mengliang Niu and Saba Hameed, the review process was handled objectively.
