# Corrigendum: Melatonin: Current Status and Future Perspectives in Plant Science

**DOI:** 10.3389/fpls.2016.00714

**Published:** 2016-05-24

**Authors:** Muhammad A. Nawaz, Yuan Huang, Zhilong Bie, Waqar Ahmed, Russel J. Reiter, Mengliang Niu, Saba Hameed

**Affiliations:** ^1^Key Laboratory of Horticultural Plant Biology, College of Horticulture and Forestry Sciences, Huazhong Agricultural University, Ministry of EducationWuhan, China; ^2^Department of Horticulture, University College of Agriculture, University of SargodhaSargodha, Pakistan; ^3^Sector Advisor-Horticulture, USAID-CNFALahore, Pakistan; ^4^Department of Cellular and Structural Biology, University of Texas Health Science Center at San AntonioSan Antonio, TX, USA

**Keywords:** melatonin, biosynthesis, physiological functions, antioxidants, root growth, stress tolerance

## Biosynthesis

The readers are informed that the text given in the biosynthesis section of originally published article (doi: 10.3389/fpls.2015.01230) at line number 20–30 of page number two is not definitive and has very limited scientific evidence, so it should not be considered.

Some steps illustrated in originally published Figure 1, like the conversion of tryptamine to Indo-3-acetaldehyde and indole acetic acid (IAA), and direct conversion of serotonin to melatonin by SNAT are not definitive, as they have very limited scientific evidence. So these steps should not be considered the part of originally published Figure 1.

**Figure 1:**
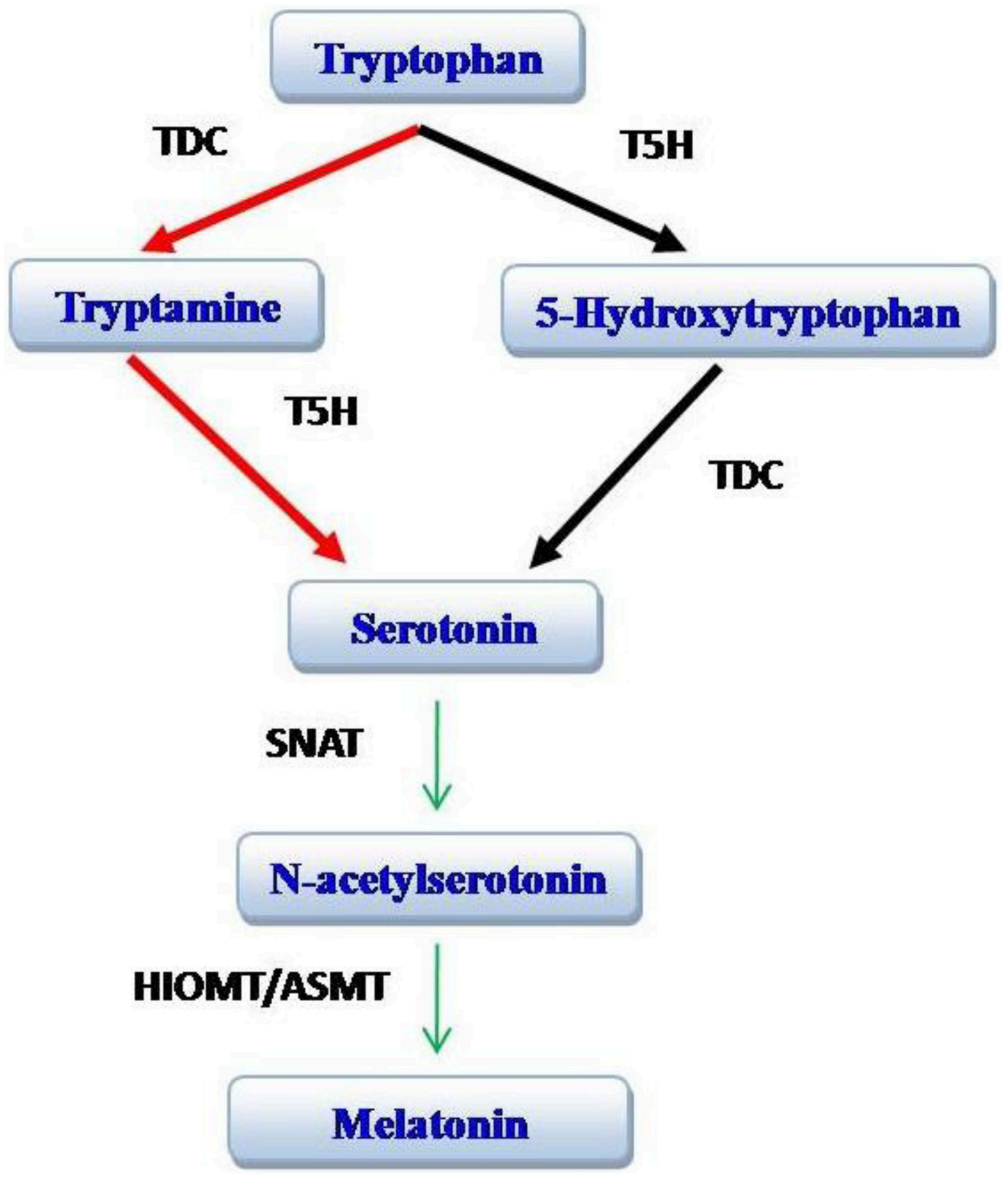
**Biosynthesis of melatonin**. The red arrows identify the preferred pathway in plants while the black arrows identify the major pathway in animals. TDC, tryptophan decarboxylase; T5H, tryptophan 5-hydroxylase; SNAT, serotonin N-acetyltransferase; HIOMT, hydroxyindole-*O*-methyltransferase [also known as acetyl serotonin methyl transferase (ASMT)]. Modified from Arnao and Hernandez-Ruiz (2014).

## Author contributions

All authors listed, have made substantial, direct, and intellectual contribution to the work, and approved it for publication.

### Conflict of interest statement

The authors declare that the research was conducted in the absence of any commercial or financial relationships that could be construed as a potential conflict of interest.
